# Ecological adaptation and phylogenetic analysis of microsymbionts nodulating *Polhillia, Wiborgia* and *Wiborgiella* species in the Cape fynbos, South Africa

**DOI:** 10.1038/s41598-021-02766-2

**Published:** 2021-12-08

**Authors:** Tiisetso Mpai, Sanjay K. Jaiswal, Christopher N. Cupido, Felix D. Dakora

**Affiliations:** 1 Department of Crop Sciences, Tshwane University of Technology, Private Bag X680, Pretoria, 0001 South Africa; 2 Department of Botany, University of Fort Hare, Alice, South Africa; 3 Department of Chemistry, Tshwane University of Technology, Arcadia Campus, Private Bag X680, Pretoria, 0001 South Africa

**Keywords:** Ecology, Microbiology, Plant sciences, Biogeochemistry

## Abstract

*Polhillia*, *Wiborgia* and *Wiborgiella* species are shrub legumes endemic to the Cape fynbos of South Africa. They have the ability to fix atmospheric N_2_ when in symbiosis with soil bacteria called ‘rhizobia’. The aim of this study was to assess the morpho-physiological and phylogenetic characteristics of rhizobia associated with the nodulation of *Polhillia*, *Wiborgia* and *Wiborgiella* species growing in the Cape fynbos. The bacterial isolates from root nodules consisted of a mixture of fast and intermediate growers that differed in colony shape and size. The isolates exhibited tolerance to salinity (0.5–3% NaCl) and pH (pH 5–10) and different antibiotic concentrations, and could produce 0.51 to 51.23 µg mL^−1^ of indole-3-acetic acid (IAA), as well as solubilize tri-calcium phosphate. The ERIC-PCR results showed high genomic diversity in the rhizobial population and grouped them into two major clusters. Phylogenetic analysis based on 16S rRNA, *atpD*, *gln*II, *gyrB*, *nifH* and *nodC* gene sequences revealed distinct and novel evolutionary lineages related to the genus *Rhizobium* and *Mesorhizobium*, with some of them being very close to *Mesorhizobium australicum*. However, the phylogenetic analysis of *glnII* and *nifH* genes of some isolates showed incongruency.

## Introduction

*Polhillia*, *Wiborgia* and *Wiborgiella* species belong to the family Leguminosae and tribes Genisteae and Crotalarieae^1–3^. They are endemic to the Cape fynbos biome, recognized as one of the richest areas of flowering plants in the world^4–6^. These legumes have bright yellow and/or white flowers, which are a major attraction for tourists^3,7^. They also contribute to the fertility of fynbos soil through N_2_ fixation with native soil rhizobia. In fact, *Polhillia brevicalyx, Polhillia pallens*, *Wiborgia sericea*, *Wiborgia tetraptera*, *Wiborgia obcordata* and *Wiborgiella sessilifolia* are reported to derive between 61 and 91% of their N nutrition from symbiotic N_2_ fixation^8^.

Some rhizobial bacteria are capable of tolerating acidic conditions, often characterised by high H^+^ concentration and the increased solubility of heavy metals and trace elements^9,10^, as well as tolerance to high soil salinity which can inhibit bacterial survival, growth and persistence^11^. Some bacteria can also solubilize P from unavailable soil P complexes for plant uptake as well as produce IAA, a hormone that is involved in root formation and root elongation for increased uptake of water and nutrients^12,13^. The identification of acid, salinity and antibiotic tolerant rhizobia with the ability to produce IAA and solubilize P is a first step to selecting rhizobia for inoculant production.

Rhizobia nodulating various Cape fynbos shrub legumes have been reported^14–17^. However, information on the microsymbionts nodulating *Polhillia*, *Wiborgia* and *Wiborgiella* species endemic to the Cape is lacking. Therefore, the aim of this study was to evaluate the morpho-physiological diversity and phylogeny of bacterial symbionts associated with the nodulation of *Polhillia*, *Wiborgia* and *Wiborgiella* species. We hypothesized that the rhizobial strains nodulating these legumes have genomic stability and were same type of rhizobial species due to the restricted habitat of these wild fynbos legumes. To test these hypotheses, the following questions were addressed (1) Which rhizobial species nodulate these wild shrub legumes? (2) What are the phylogenetic behaviours of the isolates?

## Materials and methods

### Nodule sampling and description of study sites

Root nodules were collected from *Wiborgiella sessilifolia* and *Wiborgia sericea* at Bredasdorp and Travellers Rest farm, respectively (Table 1). Due to the limited number of *Polhillia pallens* plants in the Witkoppies farm, as well as the difficulties in uprooting *Wiborgia obcordata* plants in their natural stands, mature seeds and rhizosphere soil samples were collected from their respective sites (Table 1) and used to trap rhizobia in the glasshouse. All the methods were performed in accordance with the relevant regulations and guidelines. Collecting root nodules, seeds and rhizosphere soil samples was done randomly according to plant availability at each site during the wet (July to September 2018) season.

**Table 1﻿:** Summary of plant species, sample sites and soil chemical properties.

Species	Sample site	Geographic co-ordinates	pH (H_2_0)	Soil chemical properties		
				NH_4_^+^	P	Zn
				mg·kg^−1^		
*Polhillia pallens*	Witkoppies farm	34° 33′ 53″ S 19° 59′ 43″ E	4.45 ± 0.05	0.60 ± 0.00	43.50 ± 0.50	2.84 ± 0.17
*Wiborgia obcordata*	Bushmans Kloof	32° 07′ 14″ S 19° 06′ 28″ E	4.33 ± 0.09	0.04 ± 0.01	6.00 ± 0.58	0.18 ± 0.01
*Wiborgia sericea*	Travellers Rest farm	32° 04′ 15″ S 19° 04′ 32″ E	4.47 ± 0.09	0.03 ± 0.00	8.33 ± 0.33	0.66 ± 0.03
*Wiborgiella sessilifolia*	Bredasdorp/Elim Pass	34° 37′ 58″ S 19° 49′ 399″ E	8.20 ± 0.00	0.67 ± 0.48	15.67 ± 0.33	0.41 ± 0.01

### Trapping rhizobia and their isolation

Seeds of *P. pallens* and *W. obcordata* were pre-germinated using the acid scarification method^18,19^ and transplanted into sterile sand in pots^20^. Seedlings were inoculated with their respective rhizosphere soil suspension^21^. Five replicate pots were used for each treatment. All seedlings were supplied with N-free fahraeus solution as a source of nutrients^22^. After 42 days of growth in the glasshouse, effective root nodules with a red or pinkish colour were harvested from the glasshouse-grown plants for rhizobial isolation. The root nodules obtained from the field and those from the glasshouse were surface sterilized and used to isolate rhizobia, following standard procedures^20^.

### Rhizobial authentication

Surface sterilised pre-germinated seeds of *P. pallens*, and *W. sessilifolia,* were transplanted in sterilized plastic pots (1.2 dm^3^) containing autoclaved sand. Each seedling was inoculated with 1 mL (10^7^ to 10^8^ rhizobial cells ml^−1^) of the test bacterial culture under axenic conditions. The pots with seedlings were then transported to the glasshouse and left to grow under glasshouse conditions. Three replicate pots were used for each isolate and the plants were watered twice a week with N-free ferrous nutrient solution^22^. Similarly, all the test isolates were used to also inoculate cowpea seedlings in a host-range test.

### DNA extraction and ERIC-PCR genomic fingerprinting

Genomic DNA of the rhizobial isolates grown in YMB (~1 × 10^9^ rhizobia cells ml^−1^) was extracted using a GenElute Bacterial Genomic DNA kit, according to the manufacturer’s instructions (Sigma Aldrich, USA).

Fingerprints of the rhizobial isolates were evaluated by the ERIC-PCR method performed in a 15 μl reaction mixture containing 1 μl DNA (50–80 ng μl^−1^), 7 μl 2 × My Taq PCR master mix, 1 μl each ERIC forward and reverse primers and 5 μl double distilled sterilised water.

Amplification was performed in a Thermal cycle (T100 BIORAD, USA). The primers used and the amplification conditions are shown in Table S1. The PCR-amplified products were analysed by horizontal gel electrophoresis at 100 V for 3.0 h in a 1.2% agarose gel stained with ethidium bromide (1 µg ml^–1^) in 1X TAE buffer. A standard molecular marker (GeneDirex 1 kb ladder) was included to estimate the size of the fragments. The gels were photographed under UV illumination using a gel documentation system (Geldoc^Tm^ XR + , Bio-RAD, USA).

### Cluster analysis

The banding patterns were scored directly from the gel photographs and the isolates were grouped through visual inspection of the banding. The DNA fingerprints (bands) obtained from the ERIC-PCR products was used for cluster analysis. Only distinct, well-resolved, and unambiguous bands were scored faint bands and ≤ 50 bp band sizes were excluded in the cluster analysis. A binary scoring system (1 for presence and 0 for the absence of a band) was used to generate an input matrix. This was analyzed by means of the Unweighted Paired-Group Method with arithmetic mean (UPGMA)^23^. A dendrogram was then generated from the matrix using NTSYS-Pc software^24^.

### Amplification of the 16S rRNA, housekeeping and symbiotic genes

The genomic DNA of representative rhizobial strains of different clusters in the ERIC-PCR dendrogram were amplified with primers for 16S rRNA, housekeeping (*atpD, gyrB* and *glnII*) and symbiotic (*nifH* and *nodC*) genes. Amplification was performed in a 25 μl reaction mixture containing 1 μl (50–80 ng) of genomic DNA template, 3 μl 5 × My Taq Buffer, 1 μl (10 μM) forward primer, 1 μl (10 μM) reverse primer, 0.1 μl (5U) Taq polymerase (Bioline, USA) and 18.9 μl sterile distilled water. Amplifications were performed in a Thermal cycle (T100 BIORAD, USA). The primers used, and amplification conditions are indicated in Table S1. The amplified products were separated by electrophoresis at 80 V for 1 h in a 1.2% agarose gel stained with ethidium bromide (1 µg ml^–1^) in 1X TAE buffer. Standard molecular markers (GeneDirex 100 bp and 1 kb ladders) were included to estimate the length of the fragments.

### Sequencing of the 16S rRNA, *atpD*, *gyrB*, *glnII*, *nifH* and *nodC* genes and their processing

The amplified PCR products were purified using PCR clean up kit (NEB, USA) and sent to Macrogen company, The Netherlands, for sequencing. The quality of the sequences were assessed using BioEdit 7.0.0 software^25^. Closely related species were identified using the BLAST_n_ (Basic Local Alignment Search Tool) program in the NCBI (National Centre for Biotechnology Information) server. The 16S rRNA, *atpD*, *gyrB* and *glnII*, *nodC,* and *nifH* gene sequences of the reference or type strains used in this study were retrieved from the NCBI-GenBank database. Close reference type strain sequences from the NCBI GenBank database were selected and aligned with sequences of the test strains using MUSCLE^26^, and used to construct phylogenetic trees using the MEGA 6.0 program^27^. Phylogenetic trees were generated using the P-distance method to calculate evolutionary distance^28^, and evolutional history was inferred using the Maximum likelihood method^29^ algorithm with 1000 bootstraps to allow for a strong support^30^. The MEGA 6 program was used to calculate transition-transversion-ratio to know the content of homoplasy.

### Biochemical and physiological characterization of isolates

The rhizobial isolates were grown in YMB which was adjusted to different pH levels (pH 3, 5, 9 and 10). The YMB media at pH 7 was used as a control. To screen for pH tolerance, 10 µL (≈10^8^ cells/mL) of freshly prepared broth culture of each isolate was dropped into 4 mL of freshly made broth previously adjusted to the different pH levels^20^. Thereafter, they were incubated at 28 °C for seven days with constant agitation (200 rpm) on a shaker. The pH levels of 5 and 6 were maintained with a buffer using 40 mM MES, while 30 mM HEPES was used for pH 7 and 9, and 30 mM CHES for pH 10^31,32^. After seven days of incubation, the optical density of the broth cultures was measured at 660 nm using vis spectrophotometer (7300 Jenway UK).

The phosphate solubilization test was done using double agar layer plates containing B3 media (basal layer) and tri-calcium phosphate [Ca_3_(PO4)_2_] (top layer), as described by Dabo et al.^33^. The diameter of the halo zone produced around each bacterial colony was measured and taken as indicative of P-solubilizing activity. The phosphate-solubilizing index (PSI) of each isolate was derived as the ratio of the diameter of the halo zone (R) and colony diameter (r).

A colorimetric method was used to test for IAA production by isolates in tryptophan-supplemented YMA broth, as described by Ibny et al.^13^.

To test for salt (NaCl) tolerance of the rhizobial isolates, a 20 µl volume of each matured bacterial isolate was dropped on a YMA plate containing different concentrations (0.5%, 1%, 2% and 3%) of NaCl, with 0.01% NaCl as the control^13^.

### Intrinsic antibiotic resistance

Rhizobial growth was tested in YM agar media supplemented with different concentrations of each antibiotic: streptomycin (50, 100, and 200 µg ml^−1^), kanamycin, chloramphenicol and ampicillin (25, 50 and 75 µg ml^−1^) as well as neomycin (1, 5 and 10 µg ml^−1^) with 0 µg ml^−1^ antibiotic as a control^34^. All assays were done in triplicates. Colony growth was assessed after incubation at 28 °C. Isolates showing growth in all triplicate plates were considered tolerant, and isolates which did not grow, were considered susceptible to that antibiotic concentration.

## Results

### Rhizobia isolated

The original host plants (*Polhillia pallens* and *Wiborgia obcordata*) were able to nodulate with rhizosphere soil suspensions from their respective sites of collection. (Table S2). After isolation, a total of 35 isolates were obtained, five obtained from *Wiborgiella sessilifolia,* ten from *Polhillia pallens,* five from *Wiborgia sericea*, and 15 from *Wiborgia obcordata*, (Table S2).

### Morpho-physiological characterization of rhizobial isolates

About 36% of the isolates were fast-growers which took 2 to 4 days to appear on yeast mannitol agar (YMA) plates, while the remaining isolates exhibited intermediate growth rate (Table S2). Furthermore, 94% of the isolates showed small colony size (≤ 1–2 mm diameter), 77% were non-elastic in texture and cream white in colour, while 83% showed a flat-round shape.

### Authentication and host range test of rhizobial isolates

The 35 test isolates were tested for host range under glasshouse conditions. Two isolates from *Wiborgiella sessilifolia* (TUTFWB17 and TUTFWB31) and three (TUTPP4, TUTPP8 and TUTPP10) from *P. Pallens* could nodulate their original host, due to the unavailability of *Wiborgia sericea* seeds and the very poor germination of *Wiborgia obcordata* seeds, authentication of the isolates with their original hosts was not possible. Cowpea was tested as host plant for all 35 isolates, and 86% of the isolates effectively nodulated cowpea (Table S3).

### Salinity tolerance

The rhizobial isolates differed in their response to sodium chloride concentrations. All the 35 isolates could grow in medium supplemented with 0.01% NaCl (control) as well as 0.5% and 1% NaCl. 66% and 25% of isolates tolerated 2 and 3% NaCl concentrations, respectively (Table S2). Isolates TUTPP1, TUTPP4 and TUTPP5 from *P. pallens* were susceptible to 2% NaCl, while isolates TUTFWB17 and TUTFWB31 from *W. sessilifolia*, TUTPP1, TUTPP4, TUTPP5 and TUTPP10 from *P. pallens*, TUTGWO1, TUTGWO3, TUTGWO5, TUTGWO6 TUTGWO7 and TUTGWO12 from *W. obcordata* could tolerate up to 2% NaCl, susceptible at 3% NaCl (Table S2). All isolates from *Wiborgia sericea*, tolerated up to 3% NaCl concentration.

### Acidity tolerance

The rhizobial isolates differed in their response to varying pH levels. All the isolates tested grew in YMA medium pH 7 (control), while, 51% grew well at pH 5 (Table S2). In contrast, isolates TUTGWO9, TUTGWO11 and TUTGWO15 from *Wiborgia obcordata* grew at alkaline pH 9–10 (Table S2), while 14% of the isolates tolerated a wide range of pH conditions ranging from pH 5 to pH 9, and these included isolates TUTPP3 from *Polhillia pallens*, TUTGWS2 and TUTGWS3 from *Wiborgia sericea*, TUTGWO12 and TUTGWO13 from *Wiborgiella obcordata*.

### Screening for phosphate-solubilizing bacteria (PSB)

Phosphate-solubilizing bacteria are characteristically identified by the formation of a clear halo around their colonies due to phosphate solubilization on double agar-layered plates. Out of the 35 isolates tested, 34 were able to solubilize tri-calcium phosphate, though the phosphate-solubilizing ability differed as measured by the phosphate-solubilizing index (PSI) (Table S2). Isolate TUTFWB17 from *Wiborgiella sessilifolia* recorded the largest PSI index (5.0) while isolates TUTGWO9 and TUTGWO11 from *Wiborgia obcordata* showed the least Index (Table S2). Isolate TUTGWO1 from *W. obcordata* was incapable of solubilizing P.

### Indole acetic acid production

The isolates showed marked differences in their ability to produce IAA in tryptophan supplemented YMB media. Of the 35 isolates tested, 31% (11 isolates) produced a detectable amount of IAA, which ranged from 0.51 µg ml^−1^ by TUTGWO14 from *W. obcordata* to 51.23 µg ml^−1^ by TUTPP5 from *P. pallens* (Table S2).

### Intrinsic antibiotic resistance

A number of isolates were tolerant to a wide range of antibiotics tested, namely streptomycin, kanamycin, chloramphenicol, ampicillin and neomycin (Table S2)*.* The results showed that 31, 3 and 3% of the 35 test isolates tolerated 50, 100 and 200 µg ml^−1^ streptomycin respectively. Isolate TUTPP9 from *P. pallens* was tolerant to 200 µg ml^−1^ streptomycin. However, all the isolates from *Wiborgia obcordata* were susceptible to streptomycin even at its lowest concentration of 50 µg ml^−1^. For kanamycin, 89% of the test isolates were tolerant to 25 µg ml^−1^, and 11% susceptible. Only 63 and 29% at 50 and 75 µg mL^−1^, respectively were tolerant to those concentrations of kanamycin. The results also showed that 83, 80 and 63% of the 35 test isolates were tolerant to 25, 50 and 75 µg mL^−1^ chloramphenicol, respectively. All isolates from *Wiborgia sericea,* were tolerant to 75 µg ml^−1^, while isolates TUTFWB31 from *W. sessilifolia*, TUTPP5 and TUTPP10 from *P. pallens*, TUTGWO1, TUTGWO2, TUTGWO3 and TUTGWO7 from *W. obcordata*, were susceptible to 25 µg ml^−1^ chloramphenicol. Moreover, a total of 29, 40 and 54% of the test isolates could not tolerate ampicillin at 25, 50 and 75 µg ml^−1^ concentrations respectively. All *W. sericea* isolates were tolerant to 75 µg ml^−1^ ampicillin except for isolates TUTGWS3 which was susceptible to 75 µg mL^−1^. The majority of *W obcordata* (75%) isolates were susceptible to 75 µg mL^−1^ ampicillin. However, all test isolates (100%) were resistant to 1 and 5 µg mL^−1^ concentrations of neomycin, with 43% being unable to grow at 10 µg mL^−1^ concentration.

### ERIC-PCR amplification

**Figure 1:**
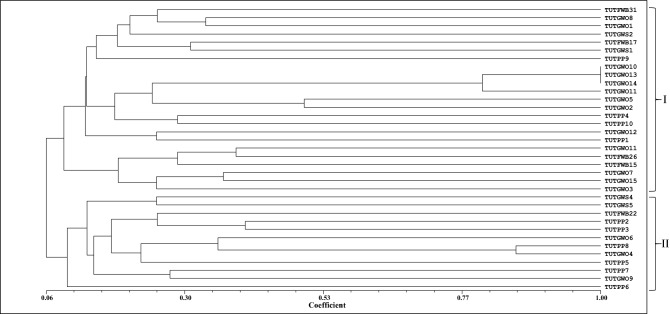
Dendrogram generated from ERIC-PCR fingerprint of 35 test isolates obtained from *Wiborgia sericea*, *Wiborgia obcordata*, *Wiborgiella sessilifolia* and *Polhillia pallens.*

PCR amplification of the ERIC region of the genomic DNA from each isolate yielded distinctive banding patterns. The dendrogram generated from the DNA fingerprints placed the 35 isolates into two major clusters (Fig. 1). Cluster I consisted of 23 isolates obtained from all the host plants with a similarity coefficient of 0.10. Isolates TUTGWO10, TUTGWO13 and TUTGWO14 from *W. obcordata* showed the highest similarity coefficients of 1.00 in Cluster I. Twelve mixed isolates from all host plants were grouped in Cluster II. (Fig. 1).

### Phylogenetic analysis of the 16S-rRNA gene

The maximum likelihood phylogeny of the 16S-rRNA gene revealed very close sequence similarities of test isolates to the genus *Mesorhizobium*.

**Figure 2:**
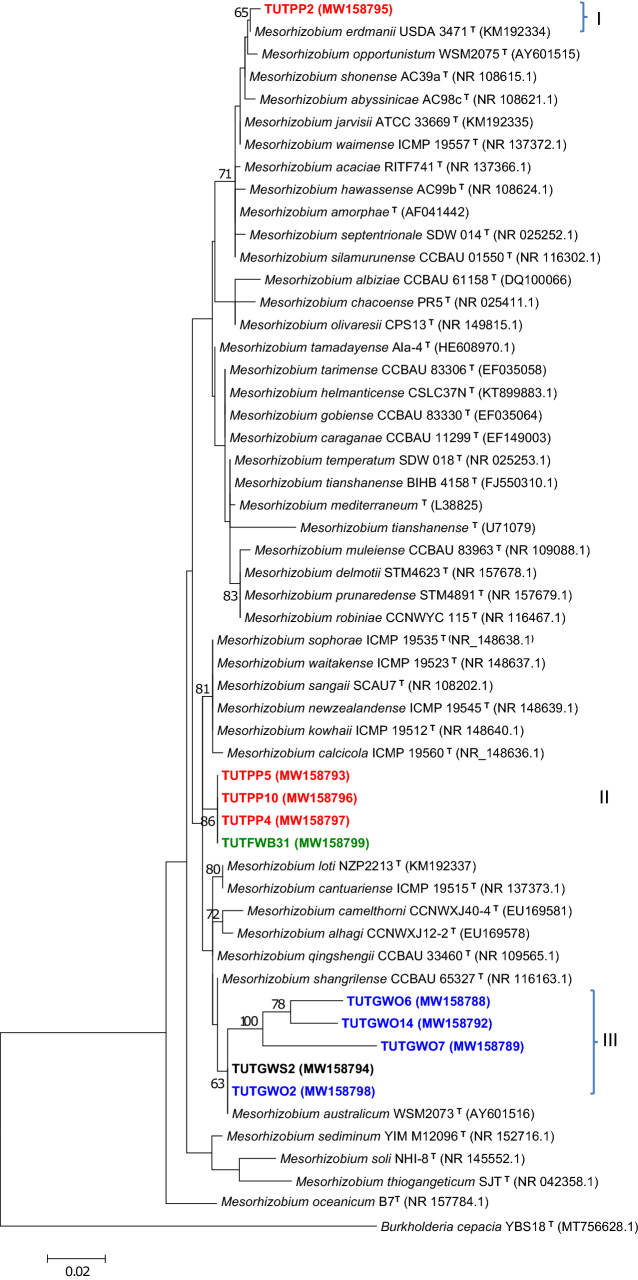
The maximum likelihood phylogenetic relationships of root nodule rhizobial isolates isolated from *Polhillia* pallens (red)*, Wiborgia obcordata* (blue), *Wiborgia sericea* (black) and *Wiborgiella sessilifolia* (green), based on *Mesorhizobium*-16S rRNA sequence analysis. Test isolates are shown in bold. The significance of each branch is indicated by a bootstrap value =  > 50 for each node (1000 replicates). The scale bar represents the number of changes per nucleotide position.

Such that*,* isolates from *Wiborgia obcordata*, *Wiborgia sericea* and *Polhillia pallens* showed close relationship with *Mesorhizobium* spp. In cluster I, isolate TUTPP2 from *P. pallens* was closely related to with *M. erdmanii* strains and shared 99.4% sequence identity, while *P. pallens* isolates TUTPP4, TUTPP5 and TUTPP10 shared 99.1% sequence identity with *M. sangaii* group as their closest relative in Cluster II. Isolates TUTGWO7, TUTGWO6, TUTGWO14 and TUTGWO2 from *W. obcordata* and TUTGWS2 from *W. sericea* revealed 95.0 to 100% sequence identity with *M. australicum* as the closest relative in Cluster III. *W. sessilifolia* isolate TUTFWB31 aligned closely with *P. pallens* isolates and together had *M. sangaii* as the closest relative with 100% sequence identity in Cluster II (Fig. 2).

### Sequence and phylogenetic analyses of housekeeping genes (*atpD*, *glnII* and *gyrB*)

**Figure 3:**
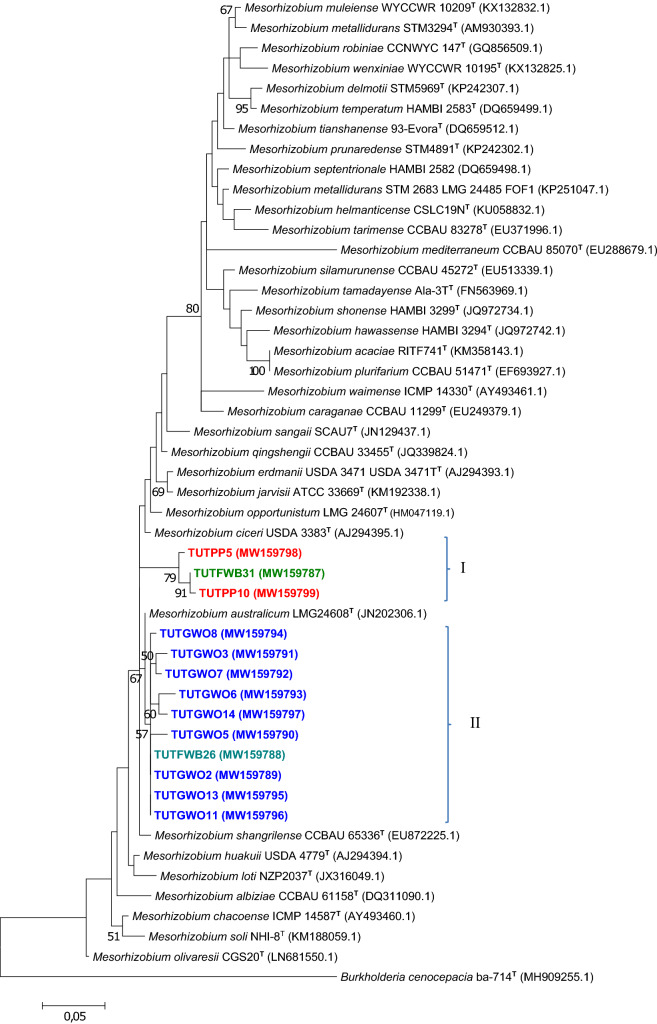
The maximum likelihood phylogenetic relationships of root nodule rhizobial isolates associated with *Polhillia pallens* (red)*, Wiborgia obcordata* (blue) and *Wiborgiella sessilifolia* (green), based on *Mesorhizobium*-*atpD* sequence analysis. Test isolates are shown in bold. The significance of each branch is indicated by a bootstrap value =  > 50 for each node (1000 replicates). The scale bar represents the number of changes per nucleotide position.

**Figure 4:**
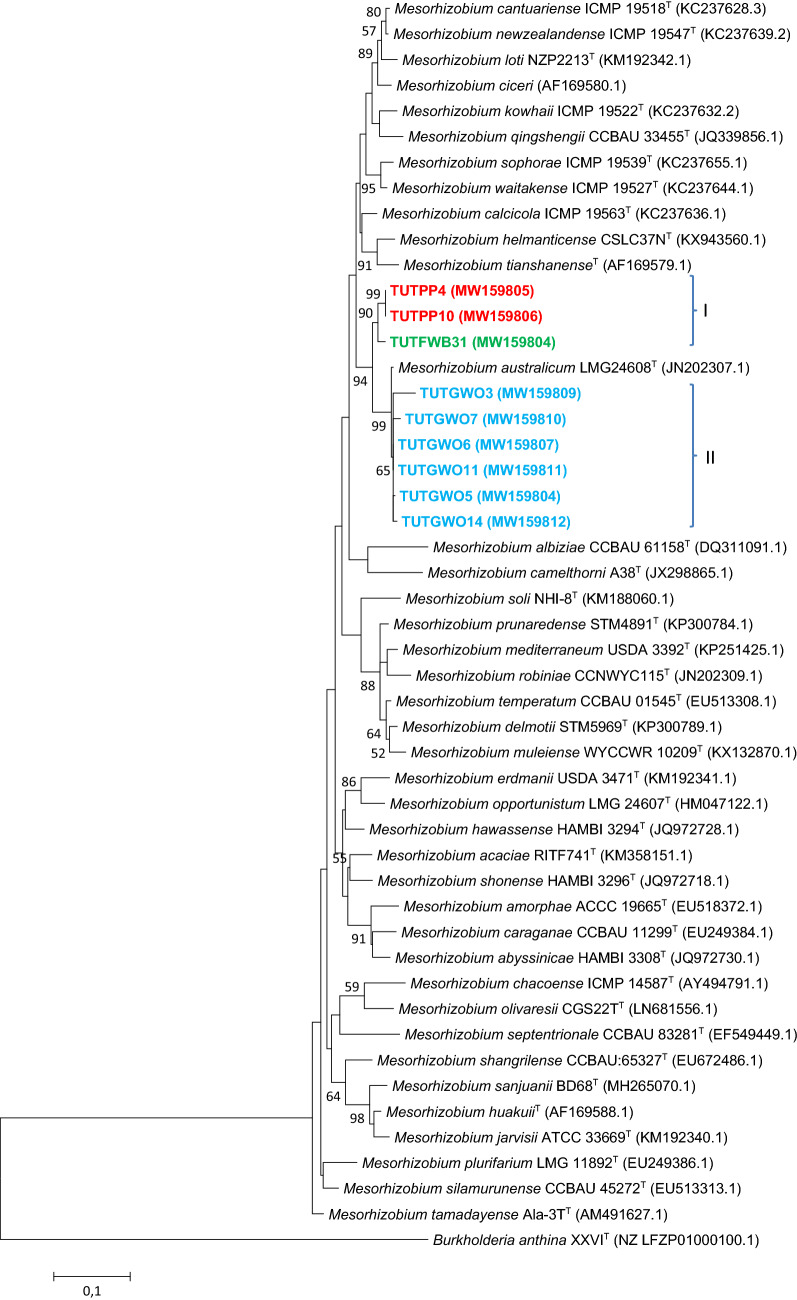
The maximum likelihood phylogenetic relationships of root nodule rhizobial isolates associated with *Polhillia pallens* (red)*, Wiborgia obcordata* (blue), and *Wiborgiella sessilifolia* (green), based on *Mesorhizobium*-*glnII* sequence analysis. Test isolates are shown in bold. The significance of each branch is indicated by a bootstrap value =  > 50 for each node (1000 replicates). The scale bar represents the number of changes per nucleotide position.

**Figure 5:**
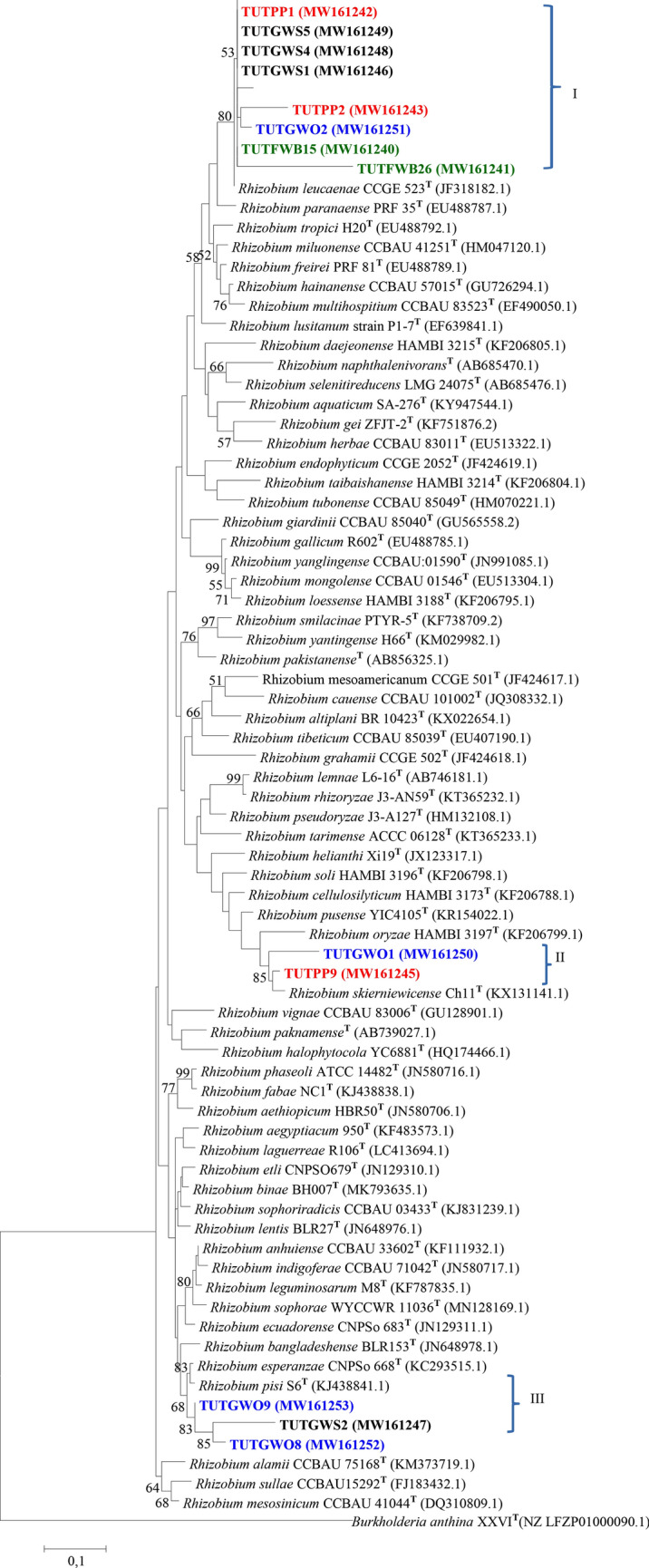
The maximum likelihood phylogenetic relationships of root nodule rhizobial isolates associated with *Polhillia pallens* (red)*, Wiborgia obcordata* (blue), *Wiborgia sericea* (black), and *Wiborgiella sessilifolia* (green), based on *Rhizobium*-*glnII* sequence analysis. Test isolates are shown in bold. The significance of each branch is indicated by a bootstrap value =  > 50 for each node (1000 replicates). The scale bar represents the number of changes per nucleotide position.

**Figure 6:**
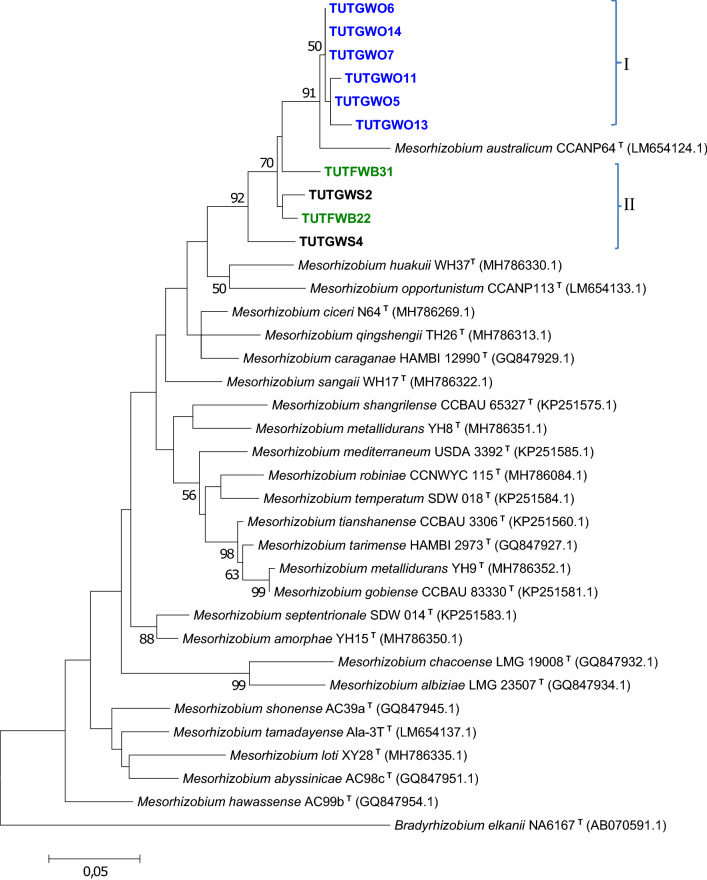
The maximum likelihood phylogenetic relationships of root nodule rhizobial isolates from *Wiborgia obcordata* (blue), *Wiborgia sericea* (blue) and *Wiborgiella sessilifolia* (green) based on *Mesorhizobium*-*gyrB* sequence analysis. Test isolates are shown in bold. The significance of each branch is indicated by a bootstrap value =  > 50 for each node (1000 replicates). The scale bar represents the number of changes per nucleotide position.

In addition to 16S rRNA, three conserved housekeeping genes (*atpD*, *glnII* and *gyrB*) were selected for phylogenetic analysis. Based on BLASTn, the isolates were placed within the *Rhizobium* and *Mesorhizobium* groups. For a clear view of the isolate groupings with reference type strains, separate phylogenies of *Rhizobium* and *Mesorhizobium* were constructed (Figs. 3, 4, 5, 6). Due to incompatibility of the primer pairs some isolates did not constantly appear in all phylogenies. Isolates from *Wiborgia obcordata, P. pallens*, *W. sericea,* and *Wiborgiella sessilifolia* occupied space in the *Mesorhizobium* trees with some discrepancies.

For example, isolates TUTGWO5, TUTGWO6, TUTGWO7, TUTGWO11 and TUTGWO14 from *Wiborgia obcordata* were aligned with *M. australicum* as the closest relative with sequence identity ranging from 97.8 (TUTGWO6) to 99.5% (TUTGWO11) in the *atpD* phylogram (Fig. 3), 98.1 (TUTGWO7) to 99.6% (TUTGWO5, TUTGWO6 and TUTGWO11 in the *glnII* phylogeny (Fig. 4), as well as 95.1 (TUTGWO11) to 95.9% (TUTWGO6, TUTGWO7 and TUTGWO14) in the *gyrB* tree (Fig. 6). Surprisingly, some isolates from *W. obcordata* aligned with *Rhizobium* in the *glnII* phylogeny. Isolates TUTGWO8 and TUTGWO9 aligned with *R. esperanzae* and respectively shared 93.2 and 97.8% sequence identity, TUTGWO1 aligned with *R. skierniewicense* and shared 90.4% sequence identity, while isolate TUTGWO2 aligned closely with *R. leucaenae* and shared 97.0% sequence identity in Clusters III, II and I respectively (Fig. 5).

Similarly, isolates from *Wiborgiella sessilifolia* which aligned with *M. australicum* appeared to be closest relative to isolate TUTFWB31 in the *atpD*, *glnII* and *gyrB* phylogenies with sequence identity of 72.8, 94.2 and 92.7% respectively. Also, isolates TUTFWB26 and TUTFWB22 had 99.5 and 93.9% sequence identity with *M. australicum* as the closest relative in the *atpD* and *gyrB* phylogenies, respectively (Figs. 3, 6). Interestingly, *glnII* sequences of isolate TUTFWB15 and TUTFWB26 aligned with *Rhizobium* spp. and recorded 99.5 and 83.4% sequence identity respectively with *R. leucaenae* as their closest relative (Fig. 5).

Isolates from *Polhillia pallens* aligned with *Mesorhizobium* in the *atpD* and *glnII* phylogenies. As found with *W. obcordata* and *W. sessilifolia* isolates, some isolates from *P. pallens* also aligned with *Rhizobium* in the *glnII* phylogeny. For instance, isolates TUTPP5 and TUTPP10 aligned together in Cluster I with *M. australicum* as their closest relative species with sequence identity of 96.0 and 95.15% in the *atpD* phylogeny (Fig. 3). Isolates TUTPP4 and TUTPP10 shared a low 84.4% sequence identity with *M. australicum* as their closest relative in the *glnII* phylogeny (Fig. 4). In contrast, isolate TUTPP9 aligned closely with TUTGWO1 from *W. obcordata* and shared 97.0% sequence identity with *R. skierniewicense* as the closest by with relative in Cluster II, while isolates TUTPP2 and TUTPP1 showed sequence identities of 92.2 and 99.5% respectively with *R. leucaenae* in Cluster I (Fig. 5).

Furthermore, the isolates from *Wiborgia sericea* aligned with *Mesorhizobium* and *Rhizobium* in the *gyrB* and *glnII* phylogenies respectively. With the *Rhizobium* phylogenies, isolates TUTGWS1, TUTGWS4 and TUTGWS5 were identical and had *R. leucaenae* as a close relative with 99.5% sequence identity in the *glnII* phylogeny (Fig. 5). Moreover, isolate TUTGWS2 had *R. esperanzae* as a closer relative species and together they shared 86.6% sequence identity in cluster III of the *glnII* phylogeny (Fig. 5). However, the sequences of isolates TUTGWS2 and TUTGWS4 aligned with *M. australicum* as their closest relative with 93.0 and 90.7% sequence identity respectively in the *gyrB* phylogeny (Fig. 6).

### Isolates’ phylogenetic position based on *nifH* and *nodC* genes

**Figure 7:**
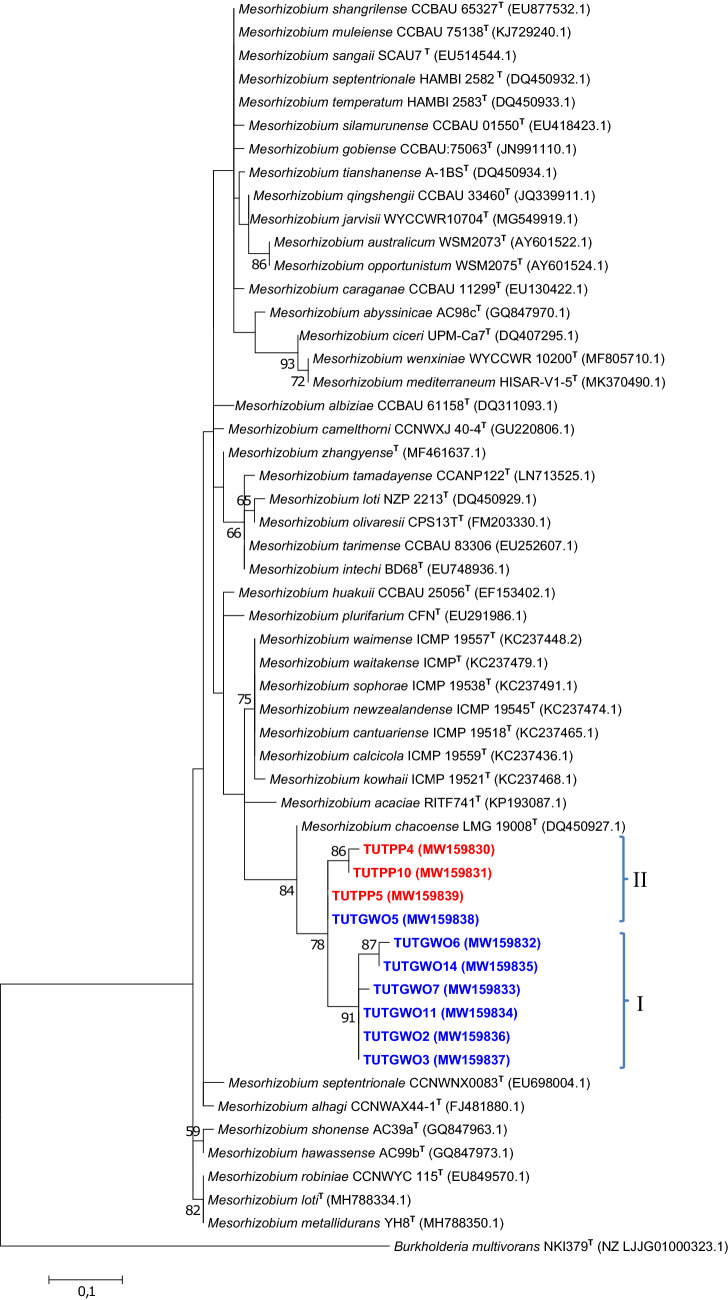
The maximum likelihood phylogenetic relationships of root nodule rhizobial isolates obtained from *Polhillia pallens* (red) and *Wiborgia obcordata* (blue) based on *Mesorhizobium*-*nifH* sequence analysis. Test isolates are shown in bold. The significance of each branch is indicated by a bootstrap value =  > 50 for each node (1000 replicates). The scale bar represents the number of changes per nucleotide position.

**Figure 8:**
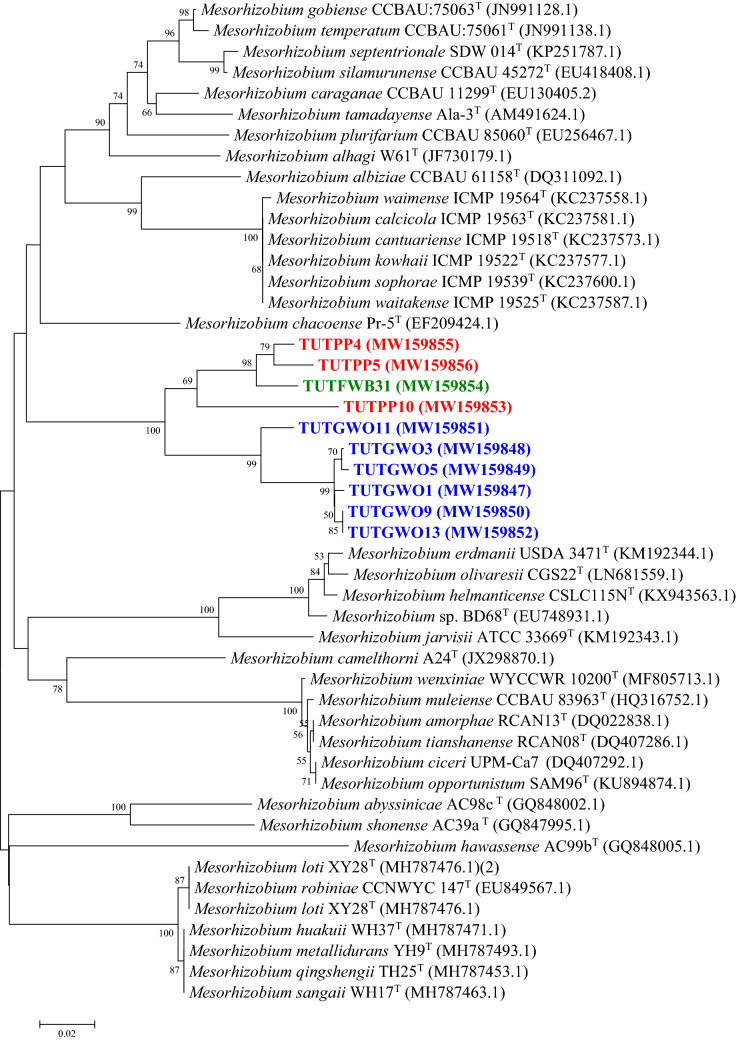
The maximum likelihood phylogenetic relationships of root nodule rhizobial isolate obtained from *Polhillia pallens* (red), *Wiborgia obcordata* (blue) and *Wiborgiella sessilifolia* (green) based on *Mesorhizobium*-*nodC* sequence analysis. Test isolates are shown in bold. The significance of each branch is indicated by a bootstrap value =  > 50 for each node (1000 replicates). The scale bar represents the number of changes per nucleotide position.

Phylogenetic analyses of *nifH* and *nodC* genes placed the test isolates closer to the *Rhizobium* and *Mesorhizobium* genera in various clusters, similar to the housekeeping gene phylograms (Figs. 7, 8), although some sequence inconsistencies between the phylogenies were observed. *Wiborgia obcordata* isolates occupied space mainly in the *Mesorhizobium* phylogeny, though some were found with *Rhizobium*. Isolates TUTGWO5 aligned closely with some *P. pallens* isolates and had 92.8% sequence identity with *M. chacoence* as the closest relative in Cluster II, while isolates TUTGWO6, TUTGWO14, TUTGWO7, TUTGWO11, TUTGWO2 and TUTGWO3 form *W. obcordata* assembled together in Cluster I and shared a low 90.5% sequence identity with *M. chacoense* as their closest relative in the *nifH* phylogeny (Fig. 7). Similarly, in the *nodC* phylogeny, *W. obcordata* isolates TUTGWO13, TUTGWO5, TUTGWO1, TUTGWO9, TUTGWO3 and TUTGWO11 aligned together and had a low relationship with the *Mesorhizobium* reference type strains as they shared between 82.5 and 85.8% sequence identity with *M. chacoense*, their closest relative in Cluster II (Fig. 8). In contrast to the results from the 16S rRNA, *atpD, glnII,* and *gyrB* phylogenies, isolates TUTGWO14 and TUTGWO9 aligned with *Rhizobium* in the *nodC* and *nifH* phylogenies respectively, where they shared 99.7% sequence identity with *R. tropici* as the closest relatives (data not shown).

But, similar to the results obtained from the 16S rRNA and housekeeping phylogenies, isolate TUTFWB31 from *W. obcordata* aligned with *Mesorhizobium* in the *nodC* phylogeny and shared 82.2% sequence identity with *M. chacoense* (Fig. 8). Similarly*, Polhillia pallens* isolates aligned with *Mesorhizobium*, and isolates TUTPP4 and TUTPP5 which aligned together in the *atpD* and *glnII* phylogenies had *M. chacoense* (Figs. 3, 4) as their closest relative and shared 90.4 and 92.8%, as well as 83.4 and 82.5% sequence identity the *nifH* and *nodC* phylogenies respectively (Figs. 7, 8).

## Discussion

### Ecological adaptation of native rhizobia to the Cape fynbos

The N_2_-fixing effectiveness of rhizobia is important for their ability to contribute N to cropping soil systems and/or the natural environment. However, this can be compromised by various biotic and abiotic factors. Thus, their adaptation to various stress factors is crucial for their survival in the rhizosphere^35,36^. In this study, 35 native rhizobial isolates from the Cape fynbos were tested for their tolerance to different levels of salinity, acidity and antibiotics commonly produced by antagonistic soil-borne microbes. The results revealed strong variations in their tolerance to these environmental factors. The Cape fynbos is generally characterized by sandy acidic soils. The rhizosphere soils from our study sites (except Bredasdorp) were quiet acidic (pH 4.3 and 5.5), which implies adaption of these isolates to the low pH soils of the fynbos^37^. It was therefore not surprising that 51% of the isolates in this study showed tolerance to low pH (pH 5), a finding consistent with the report for *Mesorhizobium*^38^ in the Cape fynbos.

It was also important to note that *Wiborgiella sessilifolia* isolates from the alkaline soils of the Bredasdorp site grew better at neutral and acidic pH 5, suggesting their ability to naturally maintain an intracellular pH of between 7.2 and 7.5 even with an external unfavourable pH^39–41^. The 19% of test isolates that tolerated both acidic and alkaline conditions (pH 5 to pH 9) closely mirrored the rhizobia reported to nodulate wild *Cajanas cajan* at pH 3 and 11 and *Acacia* species at pH 4.8 and 8.8^41^. Although alkalinity is less harmful to the survival of bacteria than acidity, it can lead to unavailability of certain essential minerals such as iron and manganese^42,43^, and thus affect plant growth and rhizobial survival. However, three isolates from *Wiborgia obcordata* which had *M. australicum* as their closest relative in the 16S rRNA, housekeeping and symbiotic gene analysis, could increase their cell division and grow well under alkaline conditions at pH 9 (TUTGWO11 and TUTGWO15) and pH 10 (TUTGWO9).

Furthermore, 72% of the 35 test isolates were tolerant to 3% NaCl concentrations, a finding consistent with an earlier report that isolates from wild legumes can tolerate high NaCl (3.5%) concentrations^44^. High pH and salinity are also a feature of deserts, such as the Thar desert of India^45–47^, and low pH is determinant for rhizobial selection by native legumes in central Brazil^48^. With climate change and the potential for an increase in irrigated crop production, soil salinity is likely to become a problem. Therefore, identifying rhizobial isolates with high salinity tolerance would be a solution for increased grain legume production. Additionally, in this study, 35% of the isolates could produce IAA at high concentrations, even higher than those reported for *Mesorhizobium* species^49^. IAA is a common by-product of L-tryptophane metabolism in several microorganisms, including rhizobia^50^, and secretion can promote plant root growth and increase nitrogen fixation via upregulation of the genes involved in carbon transport to N_2_-fixing bacteroids. Thus, N_2_-fixing rhizobia native to the sandy nutrient-poor soils of the Cape fynbos would have IAA production as an adaptation to supporting root growth of their homologous host legumes. This argument is re-enforced by the fact that the biosynthesis of IAA has been reported in species of *Burkholderia*, *Rhizobium*, *Mesorhizobium* and *Bradyrhizobium* in the Cape fynbos^16,51^.

Antibiosis or microbial warfare is common in resource-limited soils such as the low nutrients reported for the Cape fynbos. Under those conditions, soil microbes produce antibiotics that can inhibit cell growth and/or kill susceptible bacteria^52,53^. These antibiotics act by inhibiting protein synthesis and are therefore translational inhibitors to the target microbes. In this study, the antibiotic resistance of rhizobial isolates to streptomycin, kanamycin, ampicillin, chloramphenicol and neomycin was evaluated and found to differ markedly among isolates. About 37% of the isolates were susceptible to 10 µg ml^−1^ concentration of neomycin, an indication that this antibiotic was the least in limiting bacterial growth. Furthermore, 57% of the isolates in this study were susceptible to streptomycin, contrary to reports that fast-growing isolates from wild legumes are more tolerant of streptomycin^54,55^. More specifically, 15 isolates from *Wiborgia obcordata*, which were mostly related to *Mesorhizobium australicum* in the phylogenies, were susceptible to 25 µg ml^−1^ streptomycin. This indicates some vulnerability in their survival in soils that are rich in this antibiotic through inhibition of protein synthesis and translational errors in bacterial cells^56^**.**

### Phylogenetic analysis of microsymbionts nodulating *Polhillia*, *Wiborgia* and *Wiborgiella* in the Cape fynbos

In this study,similarities in isolate alignments and positions were observed in the *glnII*, *gyrB* and *atpD* phylogenies. For example, in the *Mesorhizobium* trees, the four isolates TUTGWO5, TUTGWO6, TUTGWO7 and TUTGWO11 from *W. obcordata* consistently aligned closer to *M. australicum* reference strain with sequence identity of up to 99.6%, a clear indication that *W. obcordata* is nodulated by *M. australicum* strain. Furthermore, isolates from *P. pallens* (TUTPP4 and TUTPP10), *W. sericea* (TUTGWS2, TUTGWS4) and *W. sessilifolia* (TUTFWB31 and TUTFWB22) also showed consistency in their alignment with *Mesorhizobium* reference type strains, with low sequence similarity values (≤ 97%), possibly suggesting novel species within *Mesorhizobium* genus. These results support the reports by Lemaire et al^15^ and Dludlu et al.^17^, that *Mesorhizobium* is a common and underestimated nodulator of most legumes in the Cape region, capable of competing effectively with *Burkholderia*. Further evidence is provided by earlier studies which reported *Mesorhizobium* species to be compatible with a variety of shrub legumes endemic to fynbos region^15,57–59^.

Some isolates in this study showed incongruency in phylogenies. For example, the phylogenetic analyses of *glnII* for isolates TUTGWS1, TUTGWS2, TUTGWS4, TUTGWS5 from *W. sericea*, TUTGWO9, TUTGWO8, TUTGWO1, TUTGWO2 from *W. obcordata* and TUTPP9, TUTPP1 and TUTPP2 from *P. pallens*, as well as isolates TUTFWB26 and TUTFWB15 from *W. sessilifolia*, suggest that this gene was probably transferred from *Mesorhizobium* to *Rhizobium* as it showed incongruency with 16S rRNA, *gyrB, atpD, nodC* and *nifH* phylogenies. Our results therefore agree with reports from Lemaire et al.^60^ who revealed events of horizontal gene transfer between *Rhizobium* and *Mesorhizobium* genera in the Cape fynbos region. Furthermore, our results supports the suggestion by Gogarten et al.^61^ who reported that the evidence for potential gene transfer events generally fall into two classes: (1) identification of genes with an unduly high level of similarity to genes found in otherwise unrelated taxa, and (2) genes whose phylogenetic relationships are not congruent with the relationships inferred from other genes in their respective genomes. Reports from Andrew et al.^62^ confirms HGT as a common and unrestricted process which can happen within and between bacterial genera. The disagreement of *glnII* with 16S rRNA phylogeny in this study was also reported by Turner and Young^63^. Phylogenetic analysis of the glutamine synthase gene of rhizobia can also provide strong evidence for horizontal or lateral gene transfer between different genera of rhizobia^63^. Because of possible horizontal gene transfer (or recombination) and variable mutations, single gene-based phylogenetic trees do not always reflect organismal phylogeny^64^.

The identification of *Rhizobium glnII* gene in isolates TUTGWS2, TUTGWS4, TUTGWS1, TUTGWS5, TUTGWO9, TUTGWO8, TUTGWO1, TUTGWO2, TUTPP9, TUTPP1, TUTPP2, TUTFWB26 and TUTFWB15 strongly supports the view that horizontal transfer of this gene occurred in fynbos soil. Some studies have reported that wild species of *Phaseolus* such as *Phaseolus parvulus,* and *Phaseolus pauciflorus* are nodulated by *Bradyrhizobium* species^65,66^. A few years ago, *Bradyrhizobium paxllaeri* and *Bradyrhizobium icense* were identified in Peru as novel bradyrhizobial species from root nodules of *Phaseolus lunatus*^67^. Even in Angola within Sub-Saharan Africa, bradyrhizobia were also isolated from common bean nodules^68^.

Isolate TUTGWO14 from *W. obcordata* grouped with *Rhizobium* in the *nodC* phylogeny, but with *Mesorhizobium australicum* in the 16S rRNA, *atpD*, *glnII* and *gyrB* phylogenies. This again suggests a transfer of symbiotic *nodC* gene from *Mesorhizobium* to *Rhizobium*, and thus mirrored the previous reports of the transfer of symbiotic genes between different groups of bacterial species^65,69–72^. Incongruency between the phylogenies of symbiotic (*nod* and *nif*) genes and those of chromosomal genes have been reported in a number of studies on rhizobia and has been confirmed as an indication of horizontal inheritance of the symbiosis genes^73–75^. Furthermore, a previous report from the Cape fynbos region has indicated that species within the Crotalarieae are capable of horizontal transfer of symbiosis genes between different genera of rhizobia^17^. Another study indicated has suggested that *Sphaerophysa salsula* isolates identified as *Rhizobium* using 16S rRNA gene sequences showed similar *nifH* sequences to those of the *Mesorhizobium* isolates, while a *Bradyrhizobium* isolate (16S rRNA) from *Caragana intermedia* had similar *nodC* sequence to the *Mesorhizobium* isolates^76^.

In this study, the phylogenetic incongruency found between *glnII* and the 16S rRNA, *gyr*B, *atpD, nodC* and *nifH* trees of our isolates indicates their genome plasticity and the lack of clarity in species boundaries, which together support horizontal gene transfer in the test isolates. Ochman et al.^77^ suggested that inter-specific recombination is responsible for the blurring of species boundaries, while phylogenetic incongruency documents gene transfer-mediated organismal diversification. The transfer of core and symbiotic genes between rhizobial genera adapted to local soil conditions can be the consequences of broad mutualistic relationships between test wild legumes and rhizobial genera.

## Conclusion

The morpho-genetically diverse rhizobia isolated from *Polhillia*, *Wiborgia*, and *Wiborgiella* species from the Cape fynbos region of South Africa were found to tolerate exposure to factors such as acidity, alkalinity, salinity and antibiotics. These isolates also differed in their varying abilities to solubilize P and/or produce IAA, thus suggesting varying ability to promote plant growth. In this study, *Mesorhizobium australicum* is the microsymbiont nodulating *Wiborgia obcordata*, while *Polhillia pallens*, *Wiborgia sericea* and *Wiborgiella sessilifolia* are nodulated by some possible novel *Mesorhizobium* spp.. The genomes arrangement of the test isolates indicate genetic plasticity which suggests the need to evaluate the symbiotic functioning and competitive advantage of these isolates using their homologous host plants.

## Supplementary Information

Supplementary Information.

## Data Availability

Data used in this study are available under following accession numbers. 16SrRNA (MW158788-MW158799), *atpD* (MW159787- MW159799), *glnII* (MW159804-MW159813), *gyrB* (MW159814- MW159823); *nifH* (MW159830-MW159846, MW161258); *nodC* (MW159847- MW159861).
