# Supplementary material for: Ecological adaptation and phylogenetic analysis of microsymbionts nodulating Polhillia, Wiborgia and Wiborgiella species in the Cape fynbos, South Africa
Source: Sci Rep. 2021 Dec 8;11:23614. doi: 10.1038/s41598-021-02766-2 (PMC8654865; doi:10.1038/s41598-021-02766-2)
Supplement: Supplementary file 1 — Supplementary Information. [file 41598_2021_2766_MOESM1_ESM.docx]

**Table S1:** Primers and PCR temperature profiles used for DNA amplification

| **Target Gene** | **5’-3’ nucleotide sequence** | **Temperature profile** | **References** |
| --- | --- | --- | --- |
| ERIC F | ATGTAAGCTCCTGGGATTCAC  AAGTAAGTGACTGGGGTGAGC | 5 min 95°C, 30 X (30 s 94 °C, 1 min 52°C, 8 min 65 °C), 16 min 65 °C | (Versalovic et al. 1991) |
| ERIC R |  |  |  |
| 16S rRNA F | AGAGTTTGATCCTGGCTCAG  CTTAAGGAGGTGATCCAGCC | 5 min at 95°C, 35 X (1 min at 95°C, 1 min at 55°C, 1 min at 72°C), 10 min at 72°C | (Li et al. 2007) |
| 16S rRNA R |  |  |  |
| *atpD* F | TCTGGTCCGYGGCCAGGAAG  CGACACTTCCGARCCSGCCTG | 2 min at 95^o^C, 35 x (45s at 95^o^C, 30s at 65^o^C, 1.5min at 72^o^C), 10 mins at 72^o^C | (Jaiswal et al. 2016) |
| *atpD* R |  |  |  |
| *glnII* F | AAGCTCGAGTACATCTGGCTCGACGG  SGAGCCGTTCCAGTCGGTGGTGTCG | 2 min at 95°C, 35 X (45s at 95°C, 30s at 65°C, 90s at 72°C), 10 min at 72°C | (Stepkowski *et al*. 2005) |
| *glnII* R |  |  |  |
| *gyrB* F | TTCGACCAGAAYTCCTAYAAGG  AGCTTGTCCTTSGTCTGCG | 10 min at 95°C, 35 X (30s at 94°C, 30s at 58°C, 1 min at 72°C), 10 min at 72°C | (Martens et al. 2007) |
| *gryB* R |  |  |  |
| *nifH* F | TACGGNAARGGSGGNATCGGCAA  AGCATGTCYTCSAGYTCNTCCA | 5 min at 94°C, 20 X [30s at 94°C, 30s at 65°C (-0.5°C/cycle), 90s at 72°C], 25 x (30s at 94°C, 30s at 55°C, 90s at 72°C), 10 min at 72°C | (Nzoué et al. 2009) |
| *nifH* R |  |  |  |
| *nodC* F | AYGTHGTYGAYGACGGTT C  CGYGACAGCCANTCKCTATTG | 30 s at 94°C , 40 × [30s at 94°C, 1 min at 55.4°C, 30s at 72°C], 5 min at 72°C | (Laguerre et al. 2001) |
| *nodC* R |  |  |  |
| *nodC* F | TGATYGAYATGG ARTAYTGG T  CGYGACARCCARTCGCTRTTG | 30 s at 94°C, 40 × [30s at 94°C, 1 at min 55.4°C, 30s at 72◦C], 5 min at 72°C | (Sarita et al. 2005) |
| *nodC* R |  |  |  |

**Table S2:** Morpho-physiological properties and nodulation information of the 35 test isolates associated with *Polhillia*, *Wiborgia* and *Wiborgiella* species of the Cape fynbos region

| **Isolate name** | **Host plant** | **Soil origin** | **Colony**  **Size**  **(mm)** | **Colony**  **Shape** | **Colony**  **Growth**  **(days)** | **NaCl**  **%** | **pH** | | **IAA**  **µg.ml^-1^** | **PSB** | **Streptomycin** | | |  | **Kanamycin** | | |  | **Chloramphenicol** | | |  | **Ampicillin** | | |  | **Neomycin** | | |
| --- | --- | --- | --- | --- | --- | --- | --- | --- | --- | --- | --- | --- | --- | --- | --- | --- | --- | --- | --- | --- | --- | --- | --- | --- | --- | --- | --- | --- | --- |
|  |  |  |  |  |  |  |  |  |  |  |  |  |  |  |  |  |  |  |  |  |  |  |  |  |  |  |  |  |  |
|  |  |  |  |  |  |  | **≤7** | **≥7** |  | **PSI** | 50 | 100 | 200 |  | 25 | 50 | 75 |  | 25 | 50 | 75 |  | 25 | 50 | 75 |  | 1 | 5 | 10 |
| TUTFWB15 | *Wiborgiella sessilifolia* | Bredasdorp | ≤1 | Flat, round | 2 | 3 | 5 |  | - | 4.0 | + | - | - |  | + | + | + |  | + | + | + |  | + | + | + |  | + | + | + |
| TUTFWB17 | *Wiborgiella sessilifolia* | Bredasdorp | ≤1 | Flat, round | 3 | 2 |  |  | 43 | 5.0 | + | - | - |  | + | + | - |  | + | + | - |  | + | + | + |  | + | + | + |
| TUTFWB22 | *Wiborgiella sessilifolia* | Bredasdorp | 1 | Flat, oval | 3 | 3 | 5 |  | 12 | 1.3 | + | - | - |  | + | + | - |  | + | + | + |  | + | + | + |  | + | + | + |
| TUTFWB26 | *Wiborgiella sessilifolia* | Bredasdorp | 1 | Flat, round | 4 | 3 |  |  | - | 3.4 | - | - | - |  | + | - | - |  | + | + | + |  | + | + | + |  | + | + | + |
| TUTFWB31 | *Wiborgiella sessilifolia* | Bredasdorp | 1 | Flat, round | 2 | 2 |  |  | 37 | 1.7 | - | - | - |  | + | - | - |  | - | - | - |  | - | - | - |  | + | + | - |
| TUTPP1 | *Polhillia pallens* | Uitvlucht farm | 1 | Flat, round | 2 | 1 |  |  | - | 4.0 | - | - | - |  | + | + | + |  | + | + | + |  | + | + | + |  | + | + | + |
| TUTPP2 | *Polhillia pallens* | Uitvlucht farm | 1-2 | Flat, oval | 2 | 3 | 5 |  | - | 2.0 | - | - | - |  | + | + | - |  | + | + | + |  | + | + | + |  | + | + | + |
| TUTPP3 | *Polhillia pallens* | Uitvlucht farm | ≤1 | Flat, round | 2 | 3 | 5 | 9 | - | 1.9 | + | - | - |  | + | + | + |  | + | + | + |  | + | + | + |  | + | + | + |
| TUTPP4 | *Polhillia pallens* | Uitvlucht farm | 1-2 | Dome, round | 4 | 1 |  |  | - | 2.1 | - | - | - |  | + | + | + |  | + | + | + |  | + | + | + |  | + | + | + |
| TUTPP5 | *Polhillia pallens* | Uitvlucht farm | 4 | Flat, round | 3 | 1 |  |  | 51 | 1.4 | - | - | - |  | + | - | - |  | - | - | - |  | - | - | - |  | + | + | + |
| TUTPP6 | *Polhillia pallens* | Uitvlucht farm | 1 | Flat, round | 5 | 3 | 5 |  | - | 1.6 | - | - | - |  | + | + | + |  | + | + | + |  | + | + | + |  | + | + | + |
| TUTPP7 | *Polhillia pallens* | Uitvlucht farm | 2 | Flat, round | 4 | 3 |  |  | - | 1.4 | + | - | - |  | + | + | - |  | + | + | + |  | + | + | - |  | + | + | + |
| TUTPP8 | *Polhillia pallens* | Uitvlucht farm | 1-2 | Flat, round | 4 | 3 | 5 |  | 41 | 4.8 | - | - | - |  | + | - | - |  | + | + | + |  | + | + | - |  | + | + | + |
| TUTPP9 | *Polhillia pallens* | Uitvlucht farm | 1 | Flat, round | 5 | 3 |  |  | 21 | 1.3 | + | + | + |  | + | - | - |  | + | + | + |  | + | - | - |  | + | + | + |
| TUTPP10 | *Polhillia pallens* | Uitvlucht farm | 1-2 | Flat, round | 4 | 2 |  |  | - | 1.4 | + | - | - |  | + | - | - |  | - | - | - |  | - | - | - |  | + | + | - |
| TUTGWS1 | *Wiborgia sericea* | Travellers Rest farm | 1 | Flat, round | 4 | 3 | 5 |  | 14 | 1.3 | + | - | - |  | + | + | + |  | + | + | + |  | + | + | + |  | + | + | + |
| TUTGWS2 | *Wiborgia sericea* | Travellers Rest farm | ≤1 | Flat, round | 5 | 3 | 5 | 9 | - | 4.0 | + | - | - |  | + | + | + |  | + | + | + |  | + | + | + |  | + | + | + |
| TUTGWS3 | *Wiborgia sericea* | Travellers Rest farm | 1 | Flat, oval | 6 | 3 | 5 | 9 | - | 1.2 | + | - | - |  | + | + | - |  | + | + | + |  | + | + | - |  | + | + | + |
| TUTGWS4 | *Wiborgia sericea* | Travellers Rest farm | ≤1 | Flat, round | 4 | 3 | 5 |  | - | 2.9 | + | - | - |  | + | + | + |  | + | + | + |  | + | + | + |  | + | + | - |
| TUTGWS5 | *Wiborgia sericea* | Travellers Rest farm | ≤1 | Flat, round | 6 | 3 |  |  | - | 4.0 | + | + | - |  | + | + | - |  | + | + | + |  | + | + | + |  | + | + | + |
| TUTGWO1 | *Wiborgia obcordata* | Bushmans kloof | 2 | Flat, round | 4 | 2 | 5 |  | 1 | - | - | - | - |  | + | - | - |  | - | - | - |  | + | - | - |  | + | - | - |
| TUTGWO2 | *Wiborgia obcordata* | Bushmans kloof | 2-3 | Flat, round | 3 | 3 |  |  | - | 1.5 | - | - | - |  | + | - | - |  | - | - | - |  | - | - | - |  | + | + | - |
| TUTGWO3 | *Wiborgia obcordata* | Bushmans kloof | 2 | Flat, round | 3 | 2 | 5 |  | 33 | 1.5 | - | - | - |  | + | - | - |  | - | - | - |  | - | - | - |  | + | + | - |
| TUTGWO4 | *Wiborgia obcordata* | Bushmans kloof | 1-2 | Flat, round | 3 | 3 | 5 |  | - | 1.5 | - | - | - |  | + | + | + |  | + | + | - |  | + | + | + |  | + | + | - |
| TUTGWO5 | *Wiborgia obcordata* | Bushmans kloof | 2 | Flat, round | 2 | 2 | 5 |  | 4 | 3.5 | - | - | - |  | + | - | - |  | + | + | + |  | - | - | - |  | + | + | - |
| TUTGWO6 | *Wiborgia obcordata* | Bushmans kloof | 1-2 | Flat, round | 3 | 2 | 5 |  | - | 2.5 | - | - | - |  | + | + | + |  | + | - | - |  | + | + | + |  | + | + | + |
| TUTGWO7 | *Wiborgia obcordata* | Bushmans kloof | 2 | Flat, round | 2 | 2 |  |  | - | 3.1 | - | - | - |  | + | - | - |  | - | - | - |  | - | - | - |  | + | + | - |
| TUTGWO8 | *Wiborgia obcordata* | Bushmans kloof | 2 | Flat, round | 2 | 3 |  |  | - | 2.0 | - | - | - |  | + | - | - |  | + | + | - |  | + | + | - |  | + | + | + |
| TUTGWO9 | *Wiborgia obcordata* | Bushmans kloof | 1-2 | Flat, round | 3 | 3 |  | 10 | - | 1.0 | - | - | - |  | + | + | + |  | + | + | + |  | + | + | + |  | + | + | - |
| TUTGWO10 | *Wiborgia obcordata* | Bushmans kloof | 2 | Flat, round | 3 | 3 | 5 |  | - | 2.0 | - | - | - |  | + | - | - |  | + | + | - |  | - | - | - |  | + | + | - |
| TUTGWO11 | *Wiborgia obcordata* | Bushmans kloof | 1 | Flat, round | 3 | 3 |  | 9 | - | 1.0 | - | - | - |  | + | + | - |  | + | + | + |  | - | - | - |  | + | + | - |
| TUTGWO12 | *Wiborgia obcordata* | Bushmans kloof | 1 | Flat, round | 3 | 3 | 5 | 9 | 30 | 1.75 | - | - | - |  | + | + | - |  | + | + | + |  | + | + | + |  | + | + | - |
| TUTGWO12 | *Wiborgia obcordata* | Bushmans kloof | 1-2 | Flat, oval | 2 | 2 | 5 | 9 | - | 2.0 | - | - | - |  | + | - | - |  | + | - | - |  | + | - | - |  | + | + | - |
| TUTGWO14 | *Wiborgia obcordata* | Bushmans kloof | 2 | Flat, round | 2 | 3 |  |  | 0.5 | 1.6 | - | - | - |  | + | - | - |  | + | - | - |  | + | + | - |  | + | + | + |
| TUTGWO15 | *Wiborgia obcordata* | Bushmans kloof | 1-2 | Flat, round | 2 | 3 |  | 9 | - | 1.25 | - | - | - |  | + | - | - |  | + | + | + |  | + | - | - |  | + | + | - |

Stands for – no growth, + well growth

**Table S3:** Nodulation and host range information of the 35 test isolates from *Wiborgia* spp., *Wiborgiella* spp., and *Polhillia* spp.

| **Isolate** | **Host plant** | **Soil origin** | **Original host** | **Cowpea** |
| --- | --- | --- | --- | --- |
| TUTFWB15 | *Wiborgiella sessilifolia* | Bredasdorp | - | + |
| TUTFWB17 | *Wiborgiella sessilifolia* | Bredasdorp | + | + |
| TUTFWB22 | *Wiborgiella sessilifolia* | Bredasdorp | - | + |
| TUTFWB26 | *Wiborgiella sessilifolia* | Bredasdorp | - | + |
| TUTFWB31 | *Wiborgiella sessilifolia* | Bredasdorp | + | + |
| TUTPP1 | *Polhillia pallens* | Ghwarriekop farm | - | + |
| TUTPP2 | *Polhillia pallens* | Ghwarriekop farm | + | - |
| TUTPP3 | *Polhillia pallens* | Ghwarriekop farm | - | + |
| TUTPP4 | *Polhillia pallens* | Ghwarriekop farm | + | + |
| TUTPP5 | *Polhillia pallens* | Ghwarriekop farm | - | + |
| TUTPP6 | *Polhillia pallens* | Ghwarriekop farm | - | + |
| TUTPP7 | *Polhillia pallens* | Ghwarriekop farm | - | + |
| TUTPP8 | *Polhillia pallens* | Ghwarriekop farm | + | + |
| TUTPP9 | *Polhillia pallens* | Ghwarriekop farm | - | + |
| TUTPP10 | *Polhillia pallens* | Ghwarriekop farm | + | + |
| TUTGWS1 | *Wiborgia sericea* | Travellers Rest farm | N/A | + |
| TUTGWS2 | *Wiborgia sericea* | Travellers Rest farm | N/A | + |
| TUTGWS3 | *Wiborgia sericea* | Travellers Rest farm | N/A | - |
| TUTGWS4 | *Wiborgia sericea* | Travellers Rest farm | N/A | + |
| TUTGWS5 | *Wiborgia sericea* | Travellers Rest farm | N/A | - |
| TUTGWO1 | *Wiborgia obcordata* | Bushmans kloof | N/A | + |
| TUTGWO2 | *Wiborgia obcordata* | Bushmans kloof | N/A | + |
| TUTGWO3 | *Wiborgia obcordata* | Bushmans kloof | N/A | + |
| TUTGWO4 | *Wiborgia obcordata* | Bushmans kloof | N/A | + |
| TUTGWO5 | *Wiborgia obcordata* | Bushmans kloof | N/A | + |
| TUTGWO6 | *Wiborgia obcordata* | Bushmans kloof | N/A | + |
| TUTGWO7 | *Wiborgia obcordata* | Bushmans kloof | N/A | + |
| TUTGWO8 | *Wiborgia obcordata* | Bushmans kloof | N/A | - |
| TUTGWO9 | *Wiborgia obcordata* | Bushmans kloof | N/A | + |
| TUTGWO10 | *Wiborgia obcordata* | Bushmans kloof | N/A | + |
| TUTGWO11 | *Wiborgia obcordata* | Bushmans kloof | N/A | - |
| TUTGWO12 | *Wiborgia obcordata* | Bushmans kloof | N/A | + |
| TUTGWO13 | *Wiborgia obcordata* | Bushmans kloof | N/A | + |
| TUTGWO14 | *Wiborgia obcordata* | Bushmans kloof | N/A | + |
| TUTGWO15 | *Wiborgia obcordata* | Bushmans kloof | N/A | + |

**+ = effective nodulation; - = no nodulation; N/A = not available**
